# RAC3 more than a nuclear receptor coactivator: a key inhibitor of senescence that is downregulated in aging

**DOI:** 10.1038/cddis.2015.218

**Published:** 2015-10-15

**Authors:** P N Fernández Larrosa, M Ruíz Grecco, D Mengual Gómez, C V Alvarado, L C Panelo, M F Rubio, D F Alonso, D E Gómez, M A Costas

**Affiliations:** 1 Laboratorio de Biología Molecular y Apoptosis, Instituto de Investigaciones Médicas Alfredo Lanari, IDIM-CONICET, Facultad de Medicina, Universidad de Buenos Aires, Combatientes de Malvinas 3150, Buenos Aires C1427ARO, Argentina; 2 Laboratorio de Oncología Molecular, Universidad Nacional de Quilmes, R. Sáenz Peña 352, Bernal, Buenos Aires B1876BXD Argentina

## Abstract

Receptor-associated coactivator 3 (RAC3) is a nuclear receptor coactivator usually overexpressed in tumors that exerts oncogenic functions in the cytoplasm and the nucleus. Although as part of its oncogenic actions it was previously identified as an inhibitor of apoptosis and autophagy, its expression is required in order to preserve the pluripotency and embryonic stem cell self-renewal. In this work we investigated its role in cellular senescence. We found that RAC3 overexpression in the nontumoral HEK293 cells inhibits the premature senescence induced by hydrogen peroxide or rapamycin. The mechanism involves not only the inhibition of autophagy early induced by these stimuli in the pathway to senescence, but also the increase in levels and nuclear localization of both the cell cycle suppressors p53/p21 and the longevity promoters FOXO1A, FOXO3A and SIRT1. Furthermore, we found that RAC3 overexpression is required in order to maintain the telomerase activity. In tumoral HeLa cells its activity was inhibited by depletion of RAC3 inducing replicative senescence. Moreover, we demonstrated that *in vivo*, levels of RAC3 are downregulated in the liver from aged as compared with young rats, whereas the levels of p21 are increased, correlating with the expected senescent cell contents in aged tissues. A similar downregulation of RAC3 was observed in the premature and replicative senescence of human fetal WI-38 cells and premature senescence of hepatocyte HepG2 cell line. Taken together, all these results demonstrate that RAC3 is an inhibitor of senescence whose downregulation in aged individuals could be probably a tumor suppressor mechanism, avoiding the clonal expansion of risky old cells having damaged DNA.

Receptor-associated coactivator 3 (RAC3) was originally identified as a nuclear receptor coactivator^1^ and is a member of the p160 nuclear receptor coactivator family.^2^ Although it was first described as a molecule overexpressed in breast tumors,^3^ the discovering of its role as an NF-*κ*B (nuclear factor *κ*-light-chain-enhancer of activated B cells) coactivator,^4^ together with some additional cytoplasmatic functions nonrelated to its histone acetylase activity^5, 6^ and the overexpression in a broad spectrum of tumors,^3, 7, 8, 9, 10^ made this molecule an oncogene.

Replicative senescence naturally happens to normal cells after a determined number of replicative cycles, first as a consequence of telomere shortening at each cell cycle because the mature differentiated somatic cells do not have telomerase activity.^11^ However, this irreversible arrest may also be induced by several additional stress stimuli like oncogene activation, DNA damage, a simultaneous combination of growth stimulatory signals together with cell cycle suppressors and mitochondrial effectors.^12, 13, 14, 15, 16, 17^ This last process, known as premature senescence, may be induced not only in somatic cells, but also in stem cells where the telomerase remains active.^15, 18, 19, 20^

Therefore, senescence is a process directly associated with aging.^16, 21^ In fact, aged tissues have an increased number of senescent cells and a reduced number of stem cells that maintain all their potential for self-renewal and differentiation.^15, 18, 19, 22^ The increase in senescent cells that accompanies the aging process is a consequence of damaged DNA accumulation along life because of telomere shortening, genotoxic stress and diet.^22, 23, 24, 25, 26, 27, 28^ The mammalian transcription factors FOXO (forkhead box O transcription factor) and their orthologs in *C. elegans* and *D. melanogaster* are associated with longevity.^29^ It is required for the normal response to oxidative stress and is negatively regulated by AKT phosphorylation that can be activated by insulin/insulin-like growth factor (IGF). In fact, this post-translational modification inhibits its transactivation and promotes the nuclear export. However, there are additional post-translational modifications that enhance their activity; such is the case of deacetylation by Sirtuin 1 (SIRT1).^28, 30, 31^

Senescence is a normal biological strategy that cells employ to survive, avoiding a clonal expansion of cells carrying damaged DNA, and the process is related to the loss of regenerative potential for aging tissues.^13^ Therefore, senescence, similar to apoptosis or autophagy, can all be tumor suppressor mechanisms.^13, 32, 33, 34^

In addition to our previous findings concerning the inhibitory role of RAC3 over apoptosis^5, 35^ and autophagy,^36^ some recent works have demonstrated that RAC3 expression is necessary to maintain the pluripotency and self-renewal of stem cells.^37, 38, 39^ The loss or downregulation of RAC3 is associated with differentiation of the stem cell to a mature cell type,^37^ where the telomerase activity is expected to be silenced. Although the mechanisms responsible for RAC3 overexpression in tumors are not at all clear to date, we have recently demonstrated that inflammatory response may upregulate the RAC3 gene expression.^40^ However, an overexpression of RAC3 and an active telomerase are usually found only in stem cells and tumors, but not in mature normal tissues, suggesting perhaps a relationship between both molecules associated with inhibition of senescence.

Therefore, with RAC3 being a molecule whose overexpression contributes to tumor development, inhibiting apoptosis and autophagy, we decided to investigate its probable role in cellular senescence.

## Results

### Hydrogen peroxide or rapamycin induces senescence of HEK293 cells

The mammalian target of rapamycin (mTOR) is involved in the control of growth and metabolism through several transduction signals.^24, 41, 42^ Similar to hydrogen peroxide (H_2_O_2_),^43^ depending on the drug concentration and cell type, it can induce apoptosis, senescence or autophagy.^36^ Therefore, we first investigated the dose and time required for treatment with each one of these stimuli in order to induce senescence in HEK293 cells.

**Figure 1:**
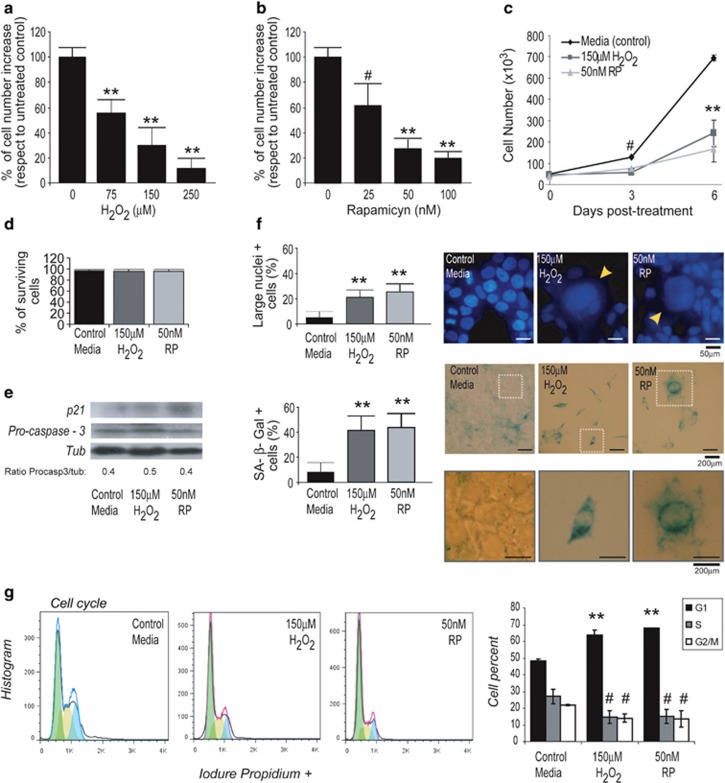
Senescence induction with hydrogen peroxide or rapamycin (Rapa) in HEK293 cells. The diagram bars show the % of cell number increase at day 6 post treatment (p.t.) with different concentrations of H_2_O_2_ (**a**) or Rapa (**b**); proliferation under control media, 150 *μ*M H_2_O_2_ and 50 nM RAPA (**c**). Cell death induction (by selected concentrations) determined by Trypan Blue staining is expressed as % of surviving as compared with control media (**d**). Absence of apoptosis and induction of senescence was determined by pro-caspase-3, caspase-3 and p21 levels by western blot (**e**) and by large nuclei+cells (arrows show large nuclei) and SA *β*-Gal+cells and expressed as percentage. Images for these positive staining are shown in right. The down panel shows the enhanced marked areas for SA *β*-Gal staining (**f**). Cell cycle analysis was performed by FACS (**g**). The diagram bars show mean±S.D. from three independent experiments. **P< 0.01, ^#^*P*<0.05; compared with control

We observed that cell proliferation under stimulation with 0.15 mM of H_2_O_2_ (Figure 1a) or 50 nM of rapamycin (Figure 1b) during 6 days was ^<^40% as compared with cells without treatment for both. After 3 days of culture in the absence of treatment, the amount of cells remained almost similar to the starting time, with a small increase at 6 days and significantly different to the growth in control cells (Figure 1c). In addition, cell survival remained >90% along all the experiment, showing no changes in cell viability (Figure 1d) as well as pro-caspase-3 levels in response to the stimuli, confirming specifically the absence of apoptosis, whereas the increase of the senescence marker p21 was clearly observed (Figure 1e).

We then analyzed the typical senescence markers, nuclei enlargement and senescence-associated *β*-galactosidase (SA *β*-Gal) induction^44, 45^ (Figure 1f). Both images and diagram bars clearly show a significant increase in cells having large heterochromatic nuclei and positivity for SA *β*-Gal activity after 6 days of H_2_O_2_ or rapamycin treatment. All these results correlate with that obtained by the cell cycle analysis, where either H_2_O_2_ or rapamycin induced the increase of G1-arrested cells (Figure 1g).

### RAC3 overexpression inhibits H_2_O_2_- or rapamycin-induced senescence

HEK293 cells normally express limiting quantities of the RAC3.^5, 36^ We investigated the effect of RAC3 overexpression in senescence.

**Figure 2:**
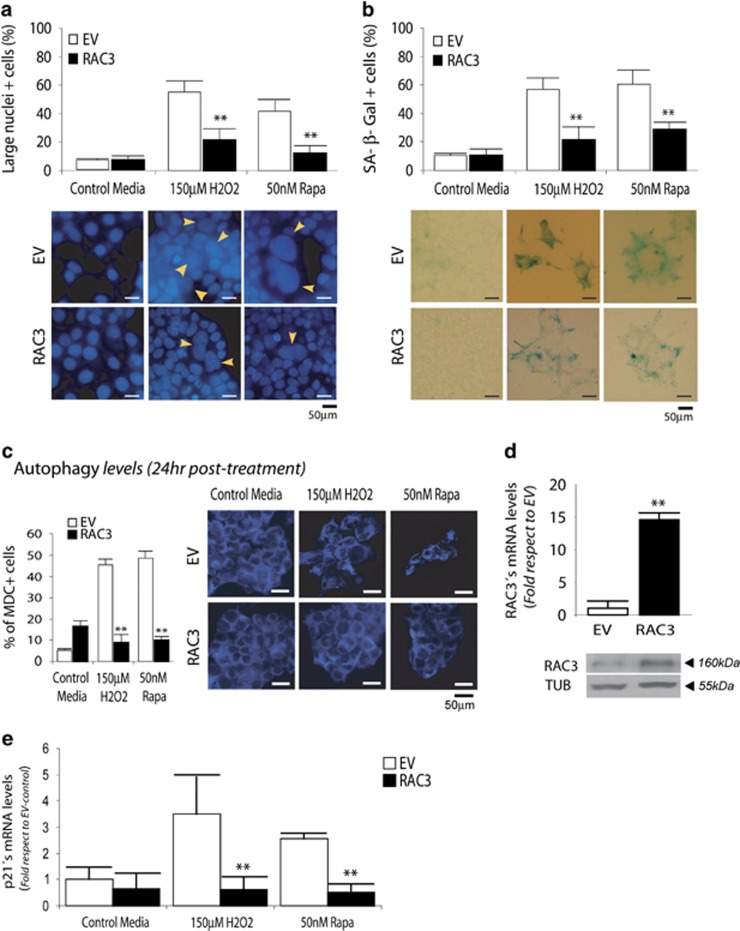
RAC3 inhibits senescence induced by hydrogen peroxide or rapamycin (Rapa) treatment. HEK293 cells, stably transfected with an empty (EV) or RAC3-expressing vector (RAC3), were incubated with 150 *μ*M H_2_O_2_ or 50 nM Rapa, and after 24 h, the media were changed until cells were collected 6 days post treatment (p.t.). Senescence is evaluated by large nuclei+cells (arrows show large nuclei) (**a**) and SA *β* -Gal+cells and is expressed as percentage (**b**). Autophagy assessed 24 h p.t. by monodansylcadaverine (MDC) staining is expressed as percentage in brackets (**c**). RAC3 overexpression was checked by western blot and qRT-PCR (**d**), and p21 modulation was determined by qRT-PCR (**e**). **P<0.01 compared with empty vector

As shown in Figure 2a, RAC3 overexpression significantly reduced the number of cells having large and heterochromatic nuclei and the positive SA *β*-Gal-stained cells induced by H_2_O_2_ or rapamycin. RAC3 overexpression by transfection was confirmed by western blot and quantitative real-time PCR (qRT-PCR; Figure 2d). Therefore, RAC3 overexpression inhibits senescence induced by both stimuli.

It was previously demonstrated that autophagy is induced during and facilitates the process of senescence.^46^ In addition, RAC3 overexpression is an autophagy inhibitor.^36^ Figure 2c shows that both H_2_O_2_ and rapamycin induced the increase of acid vesicles, compatible with autophagosomes, but RAC3 overexpression shows a clear inhibitory effect. Therefore, inhibition of autophagy could be one mechanism through which RAC3 overexpression inhibits the premature senescence.

**Figure 3:**
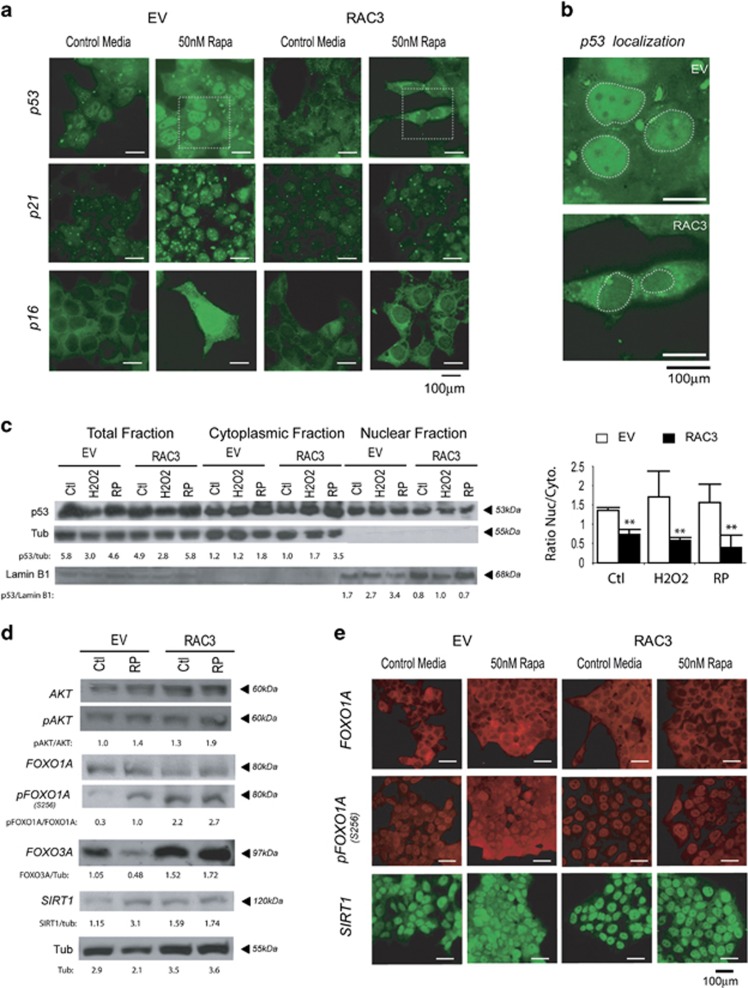
RAC3 regulates the expression and subcellular localization of senescence and aging markers. p53, p21 and p16 expression and localization were evaluated by IFI at 6 days post treatment (**a**). Enhanced image showing p53 localization in EV or RAC3-transfected cells (**b**). Total, nuclear or cytoplasmic p53 levels determined by western blot and the relative densitometric units were calculated as a ratio compared with tubulin or Lamin B1. In bars are shown mean±S.D. **P< 0.01; compared with EV (**c**). FOXO1A, pFOXO1A (s256), SIRT1 and FOXO3A expression and localization were evaluated by western blot (**d**) or IFI at 6 days post treatment for FOXO1A, pFOXO1A and SIRT (**e**)

Besides the cross-talk between autophagy and senescence, Figure 2e shows that after 6 days of treatment with rapamycin or H_2_O_2_, they induced the increase of p21 mRNA, but it was completely blocked by RAC3 overexpression. This inhibitory effect was also observed over rapamycin-induced p16 increase (Figure 3a).

Concerning p53, no significant changes in the total amount could be observed after rapamycin (Figure 3a) or H_2_O_2_ (Figure 3c) stimulation, but, in the presence of high levels of RAC3, p53 is predominantly localized at cytoplasm (Figure 3b), as confirmed by western blot (Figure 3c).

Therefore, inhibition of p53 or p53–p21 pathway activation could be additional mechanisms through which RAC3 overexpression avoids senescence.

We then investigated the role of RAC3 overexpression over the promoters of longevity, FOXOs and SIRT1.

As expected, RAC3 overexpression induces the increase of AKT activity,^5^ and it is increased by rapamycin stimulation (Figure 3d).

FOXO1A is a target of this kinase, whose phosphorylation promotes its inactivation and future degradation. In the absence of RAC3 overexpression, we observed that rapamycin treatment increases the levels of phosphorylated FOXO1A (Figure 3d: lane 2 *versus* 1), although a clear reduction of the total amount of this protein could not be detected by rapamycin treatment. However, in the presence of RAC3 overexpression, with or without rapamycin stimulation, there is a clear reduction of the total amount of FOXO1A, accompanied with its simultaneous increase in the phosphorylated form (Figure 3d: lanes 3 and 4). Although the antibody a-FOXO1A was unable to detect the native unphosphorylated nuclear form of this protein by indirect immunofluorescence (IFI), and the a-pFOXO1A mainly recognized the native phosphorylated nuclear form, Figure 3e clearly shows the rational increase of pFOXO1A and diminished amount of total protein (cytoplasmic not phosphorylated at the upper panel, plus nuclear phosphorylated at the low panel) under RAC3 overexpression.

However, SIRT1, a positive modulator of FOXO1A activity whose expression was increased by rapamycin, was also slightly enhanced (Figure 3d) and preferentially localized in the nucleus (Figure 3e) when RAC3 was overexpressed. These results correlate with that observed for FOXO3A expression, whose inhibition by rapamycin treatment was reverted by RAC3 overexpression (Figure 3d).

Therefore, our results suggest that an increased FOXO3A and SIRT1 could be mechanisms by which RAC3 overexpression contributes to inhibit senescence.

### The levels of RAC3 expression are downregulated in aging

The old tissues usually have increased number of senescent cells and this is a consequence of both the replicative and premature senescence.^22^

**Figure 4:**
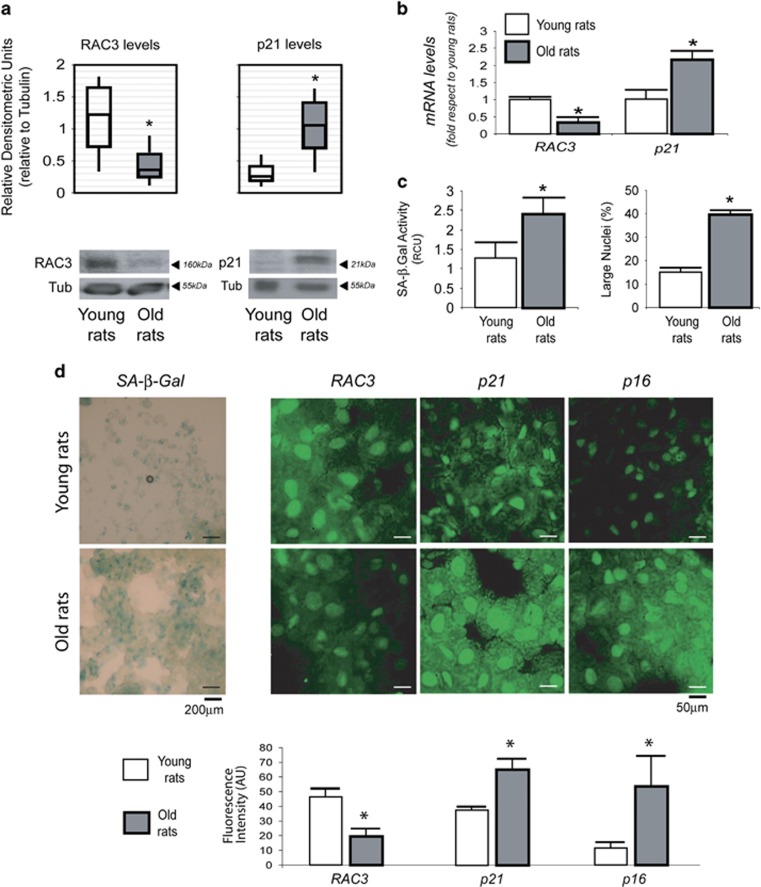
RAC3 in aging. RAC3 and p21 expression levels determined by western blot and qRT-PCR in liver from young or old rats are shown (**a**) as the relative densitometric units (**b**). The diagram bars shows the average±S.D. positive SA *β*-Gal and large nuclei in hepatocytes from young and aged rats (**c**), *P< 0.05 compared with young. Images of SA *β*-Gal hepatocytes staining and IFI of RAC3, p21 and p16 in liver sections. The diagram bars show the intensity of fluorescence as arbitrary units (AUs) determined from not less than 200 cells (**d**)

We hypothesized that perhaps RAC3 could be playing this protective antisenescent role in young individuals. In such case different levels of RAC3 expression would be expected in tissues from aged *versus* young individuals. We found that livers from old rats have a reduced expression of RAC3 as compared with the young and this is accompanied by an enhanced expression of the cell cycle suppressor p21 (Figures 4a and b) that correlates with an increased number of senescent cells as expected. Moreover, Figure 4c shows that SA *β*-Gal activity and large nuclei content were increased in aged hepatocytes, and Figure 4d shows the images for the increased SA *β*-Gal positive staining in aged hepatocytes that correlated with the increased p21 and p16 and diminished RAC3 expression detected by immunofluorescence performed in liver sections.

Therefore, although the mechanisms responsible of this change with aging are unknown, the higher levels of RAC3 in young individuals could be exerting a senescence inhibitory role that then is lost.

### RAC3 expression is downregulated in premature and replicative senescence

In order to validate all our studies concerning the role of RAC3 in premature and replicative senescence we performed additional experiments using the human fetal cell line WI-38 as model that naturally enters in replicative senescence after several passages as a primary culture. Despite this advantage, these cells are not the best model to perform stable or high efficiency transfections overexpressing RAC3 at homogeneity. However, in contrast to HEK293 cells, early passages express detectable levels of this coactivator.

**Figure 5:**
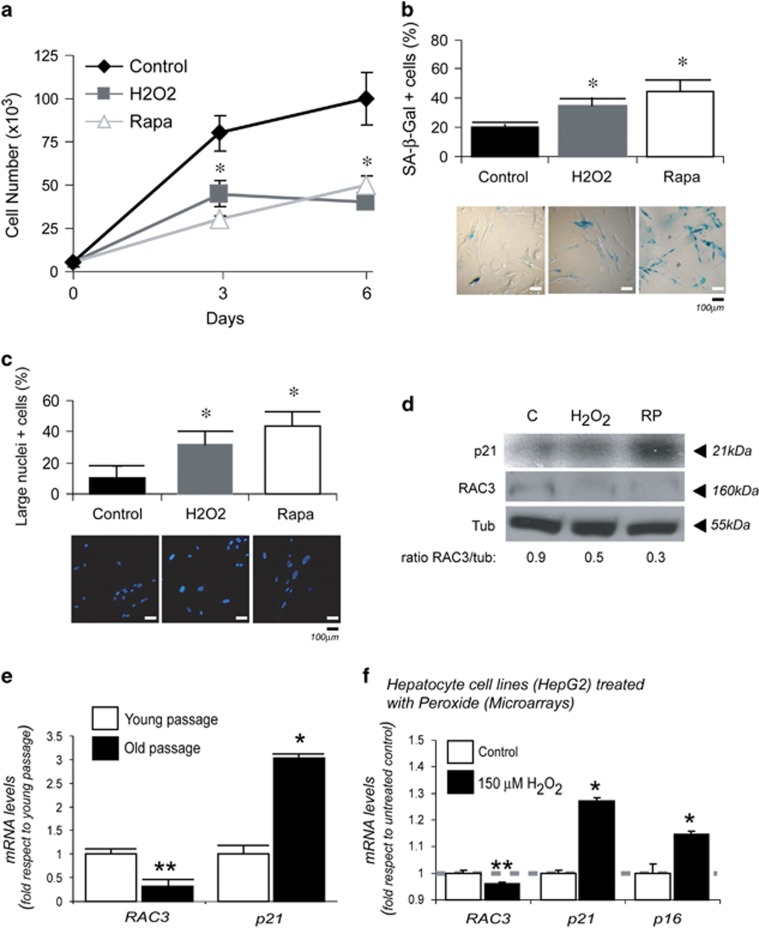
RAC3 is downregulated in premature and replicative senescence. The average from three independent experiments+S.D. of proliferation under control media, 150 *μ*M H_2_O_2_ and 50 nM rapamycin (Rapa; **a**). The diagram bars show the average+S.D. positive SA *β*-Gal (**b**) and large nuclei (**c**), *P<0.05 compared with young. RAC3 expression determined by western blot with or without Rapa treatment during 6 days (**d**). RAC3 expression in WI-38 young and old passages determined by qRT-PCR (**e**). The diagram bars show the average+S.D. of mRNA expression log-transformed values from GSE47739 data bank, platform GPL6244 Affymetrix (Santa Clara, CA, USA) (**f**). *P<0.01 and **P<0.05 compared with control

As shown in Figure 5, these cells are sensitive to rapamycin- or H_2_O_2_-induced premature senescence. After 6 days of treatment, they were clearly arrested (Figure 5a) and this correlated with an increased positive SA *β*-Gal staining (Figure 5b) and enlarged nuclei (Figure 5c). Interestingly, the increase in the senescent cell number correlated with a downregulation of RAC3 expression (Figure 5d).

In view of the fact that RAC3 is shown to be downregulated in premature senescence induced in WI-38 cells, we then investigated whether a similar downregulation could be obtained in replicative senescence of these cells. Therefore, we compared the levels of RAC3 mRNA from young *versus* old senescent passage.

Figure 5e shows that RAC3 expression was significantly downregulated in old passages, when cells undergo senescence.

These results are in agreement with that observed *in vivo*, suggesting that although aging involves the increase in the senescent cell number, it is accompanied by a downregulation of RAC3.

We then investigated whether RAC3 downregulation in senescence could be the most general phenomenon usually found in other biological models of senescence. Therefore, we performed bioinformatics analysis from the repository public microarrays data banks.^47^ We processed eight samples from the project GEO accession number GSE47739, where four of them correspond to H_2_O_2_-induced senescence of human hepatocyte HepG2 cell line (0.5 mM during 60 min) and the other four are the control cell line without treatment. Figure 5f shows the results that we obtained from these data. As shown in this figure, the differences in the mRNA expression pattern for RAC3, p21 and p16 between senescent or control cells are similar to that obtained in our models. These experiments clearly show that the increase of p21 and p16 in senescent cells is accompanied by a diminished expression of RAC3.

Therefore, RAC3 is a molecule that regulates the signaling of cell cycle suppressors and promoters of longevity, but is downregulated in senescence; therefore, their possible role avoiding the replicative senescence should not be excluded.

**Figure 6:**
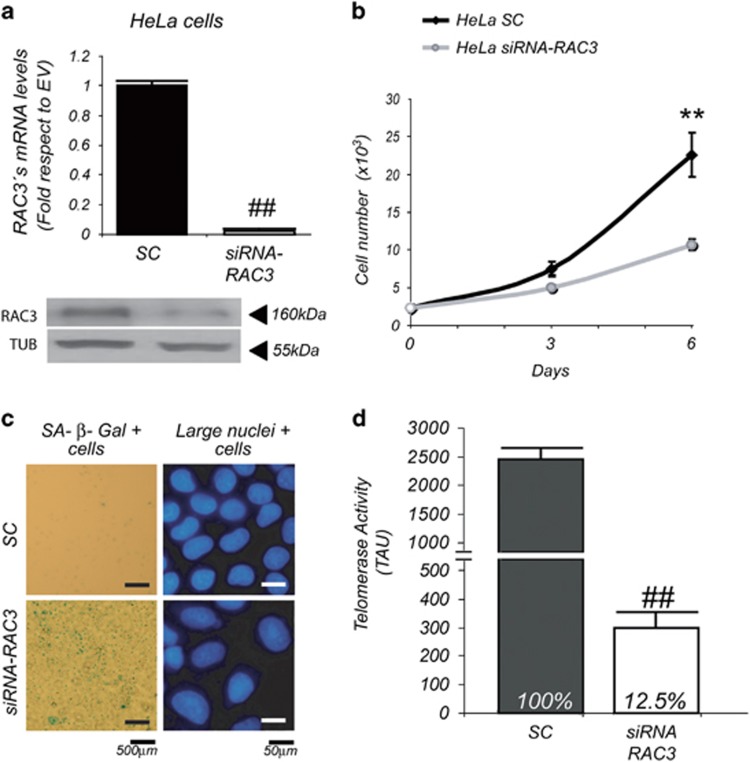
The RAC3 knocking induces the senescence of tumoral immortalized cells. Knockdown of RAC3 was confirmed by qRT-PCR and western blot in scrambled (SC)- and siRNA-RAC3-transfected HeLa cells (**a**). Proliferative response of HeLa cells transfected with siRNA-RAC3 or SC. **P<0.01, compared with SC (**b**). SA *β*-Gal activity and large nuclei images (**c**). Telomerase activity is expressed as telomerase arbitrary units (TAUs) in HeLa cells transfected with siRNA-RAC3 or SC. In bars are shown mean±S.D.; ^##^*P*<0.01; compared with SC (**d**)

While telomerase is a key factor directly involved in cell immortality that is active in stem and tumoral cells, high levels of RAC3 could be found in both cell types. Therefore, we analyzed whether RAC3 could be a required factor in order to maintain the immortality, affecting perhaps the telomerase activity. For these experiments we used a cell line having telomerase activity like HeLa, an immortal tumoral cell line that overexpresses RAC3 (Figure 6a).

Although the tumoral HeLa cells are unable to undergo spontaneous replicative senescence, we found that RAC3 knockout by transfection with a siRNA induce a diminished replicative rate as compared with control scrambled-transfected cells (Figure 6b).

Then, we analyzed a possible induction of replicative senescence induced by RAC3 downregulation. We found that this diminished replicative rate was accompanied by an increase in the typical markers of senescence: positive SA *β*-Gal activity and large nuclei (Figure 6c). In addition, the constitutive high activity of telomerase was downregulated (Figure 6d).

These experiments suggest that in this tumoral cell line the high levels of RAC3 expression are required in order to maintain its properties of perpetual growth. Although the loss of telomerase activity could be a direct consequence of the absence of RAC3 because perhaps it would be required as a positive regulator of this enzyme, another additional process affected by the absence of RAC3 that triggers senescence and downregulation of telomerase cannot be excluded.

## Discussion

We have previously shown that RAC3 is an inhibitor of both apoptosis^5, 35^ and autophagy.^36^ Although apoptosis involves cell death, autophagy not always does. In fact, the process involves first cell surviving through the elimination of damaged organelles or misfolding proteins. Something similar happens with senescence that involves an irreversible cell cycle arrest. Although this process may early contribute to avoid tumor development, it has been previously demonstrated that senescent cells are a source of several growth factors that, paradoxically, may contribute to tumor progression in the surrounding tissues.^13, 48, 49^ Indeed, the nuclear architecture of senescent cells shows some rearrangements that are related to the change in the gene expression pattern.^44^

Apoptosis, autophagy and senescence are processes involved in normal development, differentiation and all homeostatic biological responses, having a common prominent suppressing tumor development.^50^ Interestingly, in agreement with our previous findings^5, 35, 36^ and the results of the present work, RAC3 has been shown to inhibit all of these processes. Although these could be the expected activities for an oncogene, RAC3 is a special one. In fact, RAC3 does not require mutations in order to act as an oncogene. It is normally expressed at low levels in normal tissues and its tumor potential only depends upon its overexpression. RAC3 is required as a coactivator by nuclear receptors and transcription factors, and several evidences, in addition to experiments performed with knockout animals, have demonstrated that its expression is necessary for normal growth, development, metabolic and immune response.^3, 4, 40, 51, 52^ Therefore and paradoxically, although RAC3 has been defined as an oncogene, its harmful or beneficial roles are probably only dependent on its equilibrated expression levels in a specific physiological context and age.

It was recently demonstrated that RAC3 is required to maintain the pluripotent potential of stem cells.^38, 39^ In addition, it has been clearly demonstrated that this molecule is a limiting factor in all the normal tissues and overexpressed only in pathological conditions like tumors.^36^ However, despite these low expression levels in normal tissues, our results demonstrate that they are not constant and static along the life. In this regard, we have previously demonstrated that expression levels of RAC3 are under the control of the inflammatory response.^40^ Moreover, in this work we found they are downregulated in aged tissues that is accompanied by the increase of p21, a typical marker of senescence. Therefore, the loss of moderate RAC3 expression in old individuals could be a consequence of a reduction in the number and quality of somatic stem cells. In other words, perhaps the somatic stem cells are the main source of the RAC3 that is detected in western blot in young and old individuals.

Whichever the source of RAC3, our results demonstrate that it plays a role in inhibiting the premature senescence induced by both a genotoxic stimulus as H_2_O_2_ and an inhibitor of a metabolic sensor pathway as rapamycin. In agreement with our results overexpressing RAC3, its major protective effect should be found in tumoral cells, where its expression is maximal. Moreover, our findings with the tumoral cell line HeLa demonstrate that high levels of RAC3 expression are required in order to maintain its normal continuous growth and telomerase activity, whereas its loss makes this line sensitive to replicative senescence. In this regard, although it is known that HPV inactivates p53 in HeLa cells,^53^ there are evidences that p53 status is not directly associated with telomerase activity *per se* and that activation of telomerase can occur either in cells completely devoid of p53 or in cells that have functional p53.^19^ Moreover, it has also been demonstrated that repression of p53 regarding catalytic subunit of telomerase (telomerase reverse transcriptase (TERT)) expression is mediated by p21.^54, 55^ In addition, it has been previously demonstrated that the transcription of the TERT is a target of NF-*κ*B.^17, 23^ On the other hand, as we have demonstrated, high RAC3 expression correlates with p21 downregulation *in vitro* and *in vivo*, in a way that could be able to diminish TERT expression. Therefore, as changes in the expression of p53 were not evident in relation with the expression of RAC3, we could speculate that p21 inhibition by RAC3 overexpression could be a possible mechanism affecting telomerase activity, independent of p53. In addition, the inhibition of telomerase activity in the absence of RAC3 could be explained at least by the loss of the NF-*κ*B coactivator required for normal transcriptional activity of this molecule. Moreover, additional functions of RAC3 related to regulation of telomerase activity as well as telomere elongation activity independent of TERT could not be discarded.^24^ Thus, under conditions of blocked telomerase, the chromosomal damage and the consequent genetic loss can trigger senescence, even in an immortalized cell line.^56^ Although signaling through p53 has shown to be required for induction of replicative senescence after telomere shortening and DNA damage, the pathway of p16/CDK/Rb could also be involved. HeLa cells have an inactive p53, but the retinoblastoma protein (Rb) expression is normal, and therefore the antisenescent role of RAC3 could be affecting this last pathway, by inhibition of p16, thus blocking the inhibitory effect of this cell cycle suppressor over Rb inactivation.^57^

The mature differentiated cells from normal tissues do not express telomerase activity. However, moderate or high levels of this coactivator could be required in somatic stem cells, perhaps in order to preserve its telomerase activity and its pluripotent potential. Therefore, the moderate levels of RAC3 in the mature somatic cells could be preserving them from premature senescence in young cell population. We may hypothesize that RAC3 downregulation together with telomere shortening in aged individuals could be contributing to the enhanced sensitivity to diverse senescence stimuli, the increase in senescent cell number and the general failure in organs and systems that usually accompanies aging.^13, 28^ Certainly, our results suggest that RAC3 itself could be an antiaging factor. We found that it modulates the levels of molecules strongly involved in this process, promoting longevity like FOXOs and sirtuins. Both of them regulate, directly or indirectly, the expression of genes and activity of molecules related to desintoxication and longevity.^28, 30, 31^

Two models of premature senescence were analyzed in this work, using an inhibitor of the mTOR pathway as rapamycin and the oxidant H_2_O_2_ as stimuli. Our results demonstrate that RAC3 overexpression regulates the expression levels and activity of FOXOs (Figure 3).

**Figure 7:**
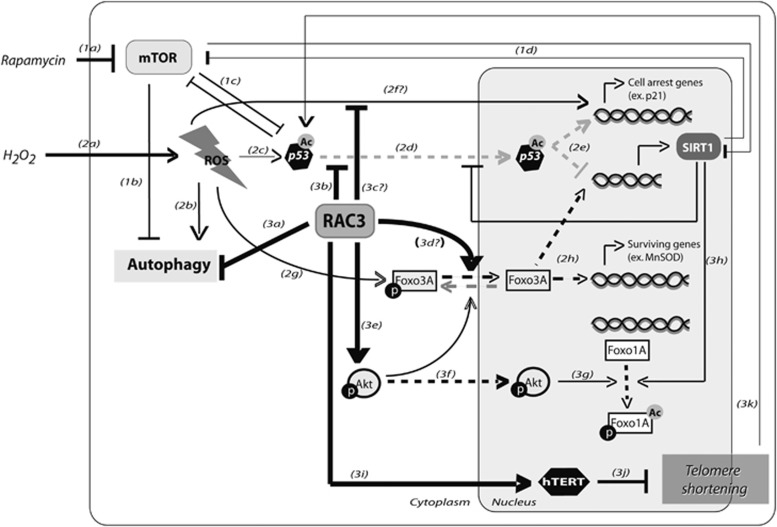
RAC3 regulates cellular senescence and aging. Rapamycin could induce senescence by inhibiting mTOR pathway (1a) that induces autophagy (1b), favors p53 transactivation (1c) and SIRT1 activity (1d). Hydrogen peroxide increases oxidative stress (2a), inducing senescence through autophagy at early stages (2b), activating p53 (2c) and its translocation from cytoplasm to nucleus (2d). Once there, it induces the expression of genes related to cell cycle arrest and senescence, like p21, in addition to apoptotic genes and represses SIRT1 expression (2e). p53-independent p21 expression could also be induced by reactive oxygen species (ROS) or mTOR inhibition by unknown mechanisms (2f). ROS also induces FOXO3A transactivation (2g), regulating positively the expression of SIRT1 and others surviving genes, like manganese superoxide dismutase (MnSOD; 2h). RAC3 inhibits autophagy (3a) and the p21 activation, involving blockage of p53 nuclear translocation (3b) or p53-independent mechanisms (3c). RAC3 allows the FOXO3A nuclear enrichment through an unknown mechanism (3d); increases AKT expression, activity (3e) and nuclear translocation (3f). Phosphorylation of FOXO1A at the serine 256 by Akt promotes its inactivation and nuclear export (3g), whereas deacetylation by SIRT1 increases its activity (3h). Finally, RAC3 could increase hTERT activity (3i) by an unknown mechanism, avoiding telomere shortening (3j) and p53 activation (3k)

In the nucleus, FOXO1A drives the transcription of genes that promote longevity.^29, 30^ However, there are at least two mechanisms that regulate its subcellular localization and activity. One of them is a negative control involving the phosphorylation by AKT at serine 256 that inhibits its DNA binding and promotes the nuclear export.^29, 30^ The other one is a positive control that increases its transcriptional activity through de-acetylation by SIRT1.^30, 31^ Although RAC3 overexpression increases the AKT activity (Figure 3), as we previously reported,^5^ and this could be suggesting a negative control over FOXO1A transcriptional activity, a simultaneous increase of FOXO3A and SIRT1 was also observed. Furthermore, SIRT1 gene is downregulated by p53 that repress its expression when it is bound to the promoter. However, when FOXO3A is localized in the nucleus, it binds p53, avoiding its inhibitory action over SIRT1 gene expression.^29^ Certainly, in this work we found that RAC3 overexpression also increases the FOXO3A expression. Therefore, taken all together, our results strongly support the antisenescence and antiaging role of RAC3 overexpression (Figure 7).

Finally, in agreement with our findings and this new concept about RAC3 as an oncogene only when it is overexpressed, its downregulation in aged individuals could probably be a tumor suppressor mechanism, avoiding the clonal expansion of risky old cells having enough damaged DNA accumulated along life. We may conclude that more than an oncogene, RAC3 could be considered a necessary molecule that under a controlled expression guarantees normal growth and development.

## Materials and Methods

### Cells and animals

The human embryonic kidney HEK293, a nontumoral cell line with low RAC3 expression, the human fetal WI-38 and the tumoral HeLa cell line, with elevated RAC3 expression, were cultured in DMEM high glucose (Gibco Laboratories, Grand Island, NY, USA) supplemented with 10% fetal bovine serum (FBS) (Natocor, Córdoba, Argentine), penicillin (100 U/ml) and streptomycin (100 mg/ml) and maintained at 37 °C in a humidified atmosphere with 5% CO_2_.

Animal experiments were performed in accordance with the Guide for the Care and Use of Laboratory Animals published by the US National Institutes of Health and approved by our Animal Ethics Committee. Female *Wistar* rats were purchased at the Buenos Aires University (Buenos Aires, Argentina) and maintained in our animal facility with food and water *ad libitum*. Livers from young rats (4 weeks old) and old rats (48 weeks old), from the same litter, were collected and used to obtain protein extracts. RAC3 and p21 expression were determined by western blot.

### Expression vectors and transfection

HEK293 cells were transfected by the CaCl_2_ method with 90% efficiency with pCMX-RAC3,^5^ or pCMX (empty vector (EV)), and stable clones overexpressing RAC3 or having limiting quantities of RAC3 were selected using 0.5 *μ*g/ml Neomycin.

HeLa cells were transfected using Lipofectamine 2000 (Natocor) with a scrambled control (SC) and the pSilencer-RAC3 plasmid (siRNA-RAC3), previously developed in our laboratory.^36^ Stable clones were selected using 0.5 *μ*g/ml Puromycin.

RAC3 expression levels were determined by qRT-PCR as previously described.^58^

### Cell culture and stimuli

Cells were plated at low density (50 000 cells/ml), and 24 h later incubated with different concentrations of H_2_O_2_ (Sigma, Buenos Aires, Argentine), rapamycin (Sigma) or control media in order to standardize the optimal concentration to induce genotoxic senescence. After 24 h, media with H_2_O_2_ or rapamycin were replaced with fresh media, and 6 days post treatment (p.t.), proliferation or senescence was evaluated.

### Proliferation and senescence

Cellular proliferation was assessed by crystal violet staining and quantification by measuring the absorbance at 570 nm of control and treated cells at days 0, 3 and 6 p.t., and results expressed as percentage of proliferation (during standardization) or in a proliferative curve (evaluated as times as compared with day 0). Senescence was determined in control and treated cells at day 6 p.t. using SA *β*-Gal assay or by Hoechst 33342 (Sigma) staining and quantification of large nuclei^44, 45^ (considering minimally three times larger than the media of the cellular population). The percentages of SA *β*-Gal+cells or large nuclei+cells were determined by counting a minimum of 200 cells per slide using fluorescence microscopy (Olympus BX51, Miami, FL, USA). Autophagy was determined in control and treated cells at 24 h p.t. using monodansylcadaverine (MDC) to detect acid vesicles as previously described.^36^

### Determination of senescence and aging-associated proteins

EV- and RAC3-transfected HEK293 cells were fixed at day 6 p.t. and immunofluorescence technique was performed using primary antibodies against p53, p21, p16, FOXO1A, pFOXO1A (ser256) and SIRT1 and their corresponding secondary antibodies, and observed by fluorescence microscopy (Olympus BX51). In case of p21 analysis, fluorescence intensity was quantified using the NIH ImageJ software (Bethesda, MD, USA) and expressed as fluorescence intensity arbitrary units. In some cases, similar experiments were performed in HeLa cells transfected with SC or siRNA-RAC3.

Cells from day 6 p.t. were also collected and total protein extract done in order to analyze by western blot. In some cases, nuclear and cytoplasmatic extracts were performed as previously described.^58^ High weight proteins (RAC3, FOXO, SIRT1) were separated in a 8% SDS-PAGE, whereas middle and low weight protein (tubulin, p53, AKT, p21) were separated in a 15% SDS-PAGE. Protein samples were blotted to a PVDF membrane. After incubation with the corresponding primary and secondary antibodies, blots were developed by enhanced chemiluminescence (New England Nuclear, Boston, MA, USA).

For all experiments, the relative densitometric units (RDUs) were determined using the NIH ImageJ software and relativized to tubulin. Tubulin was also employed to discard cytoplasmatic contamination in the nuclear extract; Lamin B1 was used to relativize the analyzed band at the nuclear extract. All antibodies were obtained from Santa Cruz Biotechnology (Santa Cruz, CA, USA), except for anti-Lamin B1 (Novus Biologicals, Littleton, CO, USA; NBP1-19804).

### Telomerase activity assay

SC- or siRNA-RAC3-transfected HeLa cell pellets were resuspended in CHAPS lysis buffer (1000 cells/ml) and incubated for 30 min on ice. After centrifugation at 16 000 × *g* for 20 min at 4 °C, aliquots of the supernatant were rapidly frozen and stored at −80 °C. Protein concentration of extracts was determined with the DC Protein Assay (Bio-Rad, Hercules, CA, USA). Telomerase activity was determined by SYBR Green RQ-TRAP assay using as a mold proteic lysates obtained. This assay is based on the protocol describe by Wege *et al.*^59^

### FACS

The assay was performed as previously described.^60^ Briefly, after different treatments, 2 × 10^6^ cells were washed twice on PBS and incubated overnight in 50 *μ*g/ml propidium iodide solution (0.1% sodium citrate, 0.1% Triton X-100) at 4 °C. Samples were analyzed in a FACSCanto II flow cytometer (BD, San Jose, CA, USA) with FACSDiva software.

### Statistical analysis

At least three independent experiments were carried out in all cases. Results were expressed as mean±S.D. The significance of differences between experimental conditions was determined using ANOVA and the Tukey–Kramer multiple comparisons test for unpaired observations.

## Abbreviations

CDK: cyclin-dependent kinase
EV: empty vector
FOXO: forkhead box O transcription factor
IFI: indirect immunofluorescence
IGF: insulin-like growth factor
MDC: monodansylcadaverine
mTOR: mammalian target of rapamycin
NF-*κ*B: nuclear factor *κ*-light-chain-enhancer of activated B cells
p.t.: post treatment
qRT-PCR: quantitative real-time PCR
RAC3: receptor-associated coactivator 3
Rb: retinoblastoma protein
RDU: relative densitometric unit
SA *β*-Gal: senescence-associated *β*-galactosidase
SC: scramble
SIRT: silent information regulator (sirtuins)
TERT: telomerase reverse transcriptase
